# Unintended Consequences of a Transition to Synchronous, Virtual Simulations for Interprofessional Learners

**DOI:** 10.3390/healthcare10112184

**Published:** 2022-10-31

**Authors:** Tiffany Champagne-Langabeer, Samuel E. Neher, Marylou Cardenas-Turanzas, Jennifer L. Swails

**Affiliations:** 1 School of Biomedical Informatics, University of Texas Health Science Center at Houston, 7000 Fannin St., Houston, TX 77030, USA; 2 McGovern Medical School, University of Texas Health Science Center at Houston, 6431 Fannin St., Houston, TX 77030, USA

**Keywords:** interprofessional care, virtual learning, medical students, nursing students, simulation, telemedicine

## Abstract

The coronavirus pandemic shifted in-person environments to virtual environments. Little is known about the effectiveness of fully synchronous, virtual interprofessional education (IPE). This study aims to compare two IPE cases that occurred in-person pre-pandemic and virtual during-pandemic. Two cases are analyzed: a medical error care and a charity care case. Participants were students from various health science disciplines. Assessments were captured through The Interprofessional Collaborative Competency Attainment Survey (ICCAS). Effect sizes were calculated for the pre-and post-surveys and analyzed using Cohen’s d for independent samples. From the in-person collection period, a total of 479 students participated in the medical error simulation and 479 in the charity care simulation. During the virtual collection period, a total of 506 students participated in the medical error simulation and 507 participated in the charity care simulation. In the data for the virtual simulations, the medical error case study maintained a large effect size (0.81) while the charity care simulation had a lesser impact (0.64 effect size). Structural details of the patient cases may be a critical variable. Future research is needed to better understand how health science students can obtain more training to notice the subtle cues from patients assessed through telemedicine modalities.

## 1. Introduction

Interprofessional education (IPE) occurs when two or more individuals from different disciplines learn together and from each other [1]. Immediate skills associated with IPE in health science disciplines include a stronger understanding of the roles other healthcare providers perform, developing communication skills, and improving teamwork skills [2,3]. In the long-term, IPE plays a significant role in delivering higher quality care to patients including improving outcomes and reducing costs [4,5]. Accrediting bodies, such as the Liaison Committee on Medical Education and the Accreditation Commission for Education in Nursing as well as Dentistry, increasingly require IPE to be included in the curriculum [6,7,8]. For IPE to be effective, learners must be active members of a team and the team should include a variety of academic backgrounds. The constructivism framework allows us to better understand how learning occurs in IPE. Constructivism, as a learning theory, states learners are active participants, and they build on previous knowledge through social interaction [9]. Learning in IPE occurs when students collaborate with and learn from other participants to solve a real-world scenario, with expert faculty available to facilitate and guide them. Studies over time highlight the skills associated with high-quality IPE have been shown to increase the quality of patient care and decrease medical costs [2,10,11,12].

Coordinating IPE activities for students is a complex process given that learners are enrolled in different schools, and students and faculty in the health science disciplines have rigorous and demanding schedules. A recognized problem with IPE is having it accessible to large amounts of students in a safe environment [13]. In 2019, the coronavirus pandemic (COVID-19) paused the public gathering of many large classes and created dramatic changes in medical education [14]. With physical distancing known to be an effective way to slow the spread of the virus, many educational activities quickly shifted to virtual settings [15]. The American Medical Association provided guidance for educators in the “Telehealth Clinical Education Playbook” [16]. Key tenets in the playbook include innovation, logistics, and skills training. Although there are several positive aspects that have emerged from the transition to virtual simulations in various settings, such as reducing barriers for faculty and students related to travel time, work–life balance, and geographic distance between educational and clinical sites; there have also been challenges. From an educator’s perspective, sourcing the appropriate videoconferencing technology may present challenges when training very large groups of students. Depending on the subject matter, student population, and institutional policies, the technology is often required to be compliance with extra security measures, passwords, and recording capabilities. In person participants can easily be separated into separate rooms to allow for smaller simulations and debriefs; however, coordinating multiple break-out rooms in virtual environments requires specialized training and additional administrative monitoring.

Noting the benefits and challenges, educators are examining hybrid approaches which combine both in-person and virtual experiences to virtual only and are looking to the future of simulation for training. In this research, we examined the impact of changing in-person educational activities to a virtual setting on a large group of students across educational disciplines. We conducted a quasi-experimental study using a retrospective pre-test and post-test design. We conducted a quasi-experimental study using a retrospective pre-test and post-test design. This type of design was selected because randomized of students would have denied a critical educational opportunity to our students [17]. The goal was to determine which format best achieves the goals of IPE and which format should be used in the future. This study will describe these findings and is relevant to academic clinicians and healthcare educators responsible for planning and executing large student simulations.

## 2. Materials and Methods

### 2.1. Case Content

The content of the case studies was created by a team of interprofessional faculty and was designed to elicit the strengths of various student roles (e.g., medical, nursing, dentistry, public health, and clinical informatics). There were two case-based scenarios, one focused on a medical error and one focused on a charity clinic in the academic years 2019–2020 and 2020–2021. The goals of the case-based encounters included improved communication, teamwork, and understanding of the roles of other healthcare providers. Students were given information about the patient including name, setting, chief complaint, and vital signs. Standardized patients played the roles of the patients and family members. The case involving a medical error started with the student teams performing a root cause analysis, and then entering a patient room with the task of disclosing a medical error. The patient—under the initial assumption they were to be discharged, reacts powerfully upon hearing the change in status. The case involving the charity care clinic began with a geriatric team huddle regarding an elderly patient who has recently lost her job and her insurance and complains of back pain. Upon entering the room, the students receive results from the radiology department that the patient has a severe spinal cord injury requiring immediate transfer to the emergency room and must make a rapid decision. In each case, the students were required to work as a team to form a mental model, support each other, and solicit feedback from respective team members. Standardized patients were trained to portray realistic patient interactions, provide intense emotional responses, and provide qualitative feedback to students after the simulations.

### 2.2. Participants

Student learners were from an academic medical center in a large, metropolitan region in Texas, USA. The medical students were in their fourth year of training; nursing students were from both undergraduate and graduate (post-baccalaureate) programs; dental students were in their third year of training, and public health and clinical informatics students were enrolled in graduate (post-baccalaureate) programs. Participation in both simulations was required for students from the medical, nursing, and dental schools before graduation and was voluntary for students from public health and clinical informatics disciplines. The Institutional Review Board of the university approved this study as quality improvement (HSC-MS-19-0362). Informed consent was obtained from all subjects involved in the study. All student data were deidentified for this study.

### 2.3. Survey Instrument

The Interprofessional Collaborative Competency Attainment Survey (ICCAS) survey was used to assess participants’ attainment of teamwork skills [18,19] Supplement File S1. The ICCAS survey is a tool used specifically to assess educational interventions within the interprofessional domain. This tool measures a defined theoretical framework of competencies and has been validated and widely used in the training of physicians, nurses, dentists, pharmacists, social workers, and other allied health professionals [20,21]. It consists of 20 questions which are provided to students only two times: before the educational intervention, and after the educational intervention. The questions evaluate a self-report of competencies about interprofessional care in education programs. Competencies measured include effective communication, roles and responsibilities, collaboration, patient-family-centered approach, conflict management, and team functioning. Items are scored on 7-point Likert scale (strongly disagree = 1 to strongly agree = 7).

### 2.4. Data Analysis

Students were enrolled in an online course for both periods (2019–2020; 2020–2021) through a learning management system (LMS) for survey data collection. Data were collected and analyzed from two different periods. The first collection period was from August 2019 to May 2020 when students participated in the educational simulations in person. The second collection period occurred from August 2020 to May 2021 when the educational simulations had transitioned to synchronous, virtual videoconferences. Students were surveyed once before the simulation and then a second time after the simulation. (The complete simulation included the event, a faculty debrief, and a time for reflection.) All survey data were collected through the LMS.

We reported frequencies, percentages, and for continuous variables measures of central tendency. We conducted Pearson’s χ square to compare categorical variables and Student’s *t*-test for continuous variables. A participant’s total score for each survey was the sum of values to each question with a possible range of 1 to 140.

We analyzed differences between the pre-survey vs. the post-survey means scores of the four study groups conducting Student’s *t*-test for independent samples and reported the mean and standard deviation. For each study group, we evaluated the Cohen’s d (95% CI) [22] as a measure of effect size. We did not expect to observe bias by using this measure, also known as “uncorrected effect size,” due to our large sample size [23]. We applied the following formula:

$$\mathrm{Cohens}’d=\frac{\chi 1-\chi 2}{s*}$$

$$\mathrm{where}\;s\;*\;=\frac{\sqrt{\left({𝓷}_{1}-1\right){s_{1}}^{2}+\left({𝓷}_{2}-1\right){s_{2}}^{2}}}{{𝓷}^{1}+{𝓷}^{2}\;-\;2}$$

We also reported the percentage of post-surveys means above the mean of the pre-survey mean for each study group, the probability of superiority defined as the chance that a student picked at random from the post-survey score group would have a higher score than a student picked at random from the same pre-survey score group, the percentage of overlap of the pre and post-survey scores, and the number of students needed to receive the simulation in order to observe a favorable outcome in the post-survey group [24].

The study outcome measure was the effect sizes of the interventions by setting (in-person or virtual) and by the type of simulation (medical error or charity care).

We reported the proportion of students by professional school affiliation, gender, and age for each case-based scenario and for the in-person and the virtual simulations. The pre-survey score was utilized for the control group, and the post-survey score was for the experimental group. The magnitude of the effect sizes was determined as the following: a small relative size was established at 0.2 effect size, meaning 58% or less of the pre-survey results were below the post-tests; a medium relative size was set at 0.5 effect size, meaning 69% of the pre-tests were below the post-tests; and a large relative size was established at 0.8 effect size, indicating 79% of the pre-tests were below the post-tests. Thresholds to determine effect sizes have been arbitrarily set and have been the source of debate for many years among researchers in many fields; however, we adopted a conservative approach by using the thresholds proposed by Cohen, J [22]. We considered *p*-values < 0.05 (two-tail) as statistically significant and conducted all analyses with Stata IC version 15, College Station, TX, USA.

## 3. Results

A total of 4057 (100%) surveys were received and after exclusion of duplicates and incomplete surveys 3942 (97.16%) surveys remained corresponding with 1971 pairs of unique participants with pre and post-tests surveys. Both simulations in person, had a total of 479 participants each, while the medical error virtual simulation had 506 participants and the charity care virtual simulation had 507 participants.

**Figure 1:**
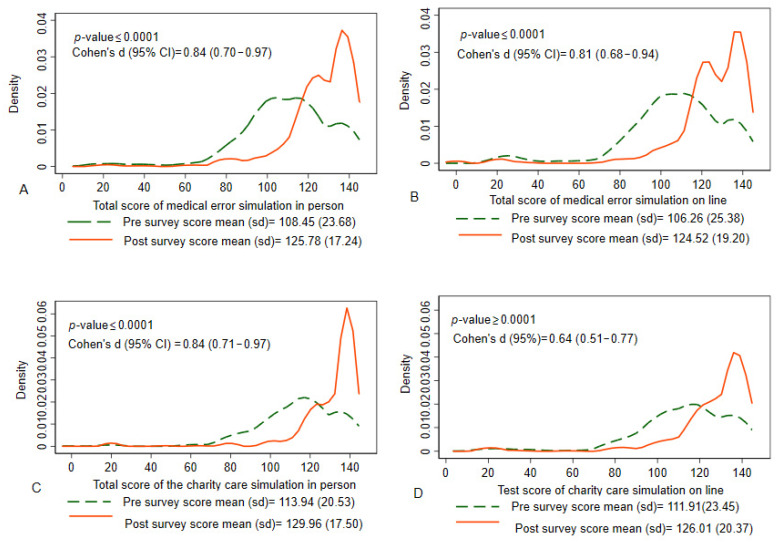
The density plots of the pre-survey and post-survey scores with mean (sd) and by the type of simulation, the Medical Error simulation in person and online (See (**A**,**B**)) and the Charity Care simulation in person and online (See (**C**,**D**)). Note: The figures show similar magnitude of effect for the medical error simulation in person and online, however when compared to the charity care simulation in person, the magnitude of effect decreases for the charity care simulation online.

A total of 479 students participated in the medical error simulation and 479 in the charity care simulation during the first collection period (August 2019–May 2020), representing the in-person results. A majority were female for both simulations (67.2%, 69.5%, respectively) and most students (86.2%, 74.1%) were in the 20–29-year age range. Students were represented from the medical school (41.5%, 43.8%), the nursing school (38.4%, 38.4%), and the remaining were accounted for by dental and public health. See Table 1. Regardless of the medical error simulation, the pre-survey mean (sd) score was 108.4 (23.68) and the post-survey mean (sd) score was 125.78 (17.24) (*p*-value ≤ 0.0001). The pre-survey and post-survey mean (sd) scores for the charity care simulation were 113.94 (20.53) and 129.96 (17.50), respectively (*p*-value ≤ 0.0001). See Figure 1A,C.

From the second collection period (August 2020–May 2021), during which time the students were engaged online only, a total of 506 students participated in the medical error simulation and 507 participated in the charity care simulation. Similar to the in-person simulations, the majority of students were female (63.0%, 62.5%) and in the 20–29 year age range (84.9%, 73.3%). The distribution of students by school was also similar to the in-person simulations, with the majority coming from the medical (37.9%, 39.0%) and nursing (34.1%, 33.5%) schools. See Table 2. In the medical error simulation online, we observed a pre-survey mean (sd) score of 106.26 (25.38) and a post-survey mean (sd) score of 124.52 (19.20), (*p*-value ≤ 0.0001). The pre- and post-surveys mean (sd) scores found in the charity care simulation were 111.91 (23.45) and 126.01 (20.37), respectively (*p*-value ≤ 0.0001). See Figure 1B,C.

The Cohen’s d was analyzed, comparing cases that were conducted in person (medical error and charity care) and resulted in a large effect size (0.84). See Table 3.

However, when analyzing the data for the virtual simulations, only the medical error resulted in a large effect size (0.81) while the charity care simulation had a medium effect size (0.64).

## 4. Discussion

This study examined the changes in student learning at one large academic medical center as learners transitioned from an in-person simulation to a virtual simulation over a two-year period in healthcare programs of institutions located in the United States. The outcomes are valuable as complex education programs determine their future model of interacting with students, whether it is necessary to remain virtual, or if options exist to return to fully in-person instruction or a hybrid approach. Our results indicate it is possible to successfully transition a large interprofessional program from in-person to virtual and maintain an educational experience that is feasible for faculty administrators and results in positive changes in student scores. Our results, however, show an unintended consequence of a decreased effect size of one of the case studies used with the students when moving to a virtual format. The case study focused on a charity clinic with an elderly patient had a large effect size when delivered in person; however, the impact on student learning was less pronounced (medium impact) when delivered virtually. Our faculty team has considered multiple reasons for this discrepancy. In the charity care case, the standardized patient is portrayed as an older woman who is experiencing dental pain. She is depressed, has limited body language, and is soft-spoken. In our second case—the medical error case, the male patient is angry, has significant body language, and yells at the students for causing damage to his body. The students are often expressive afterward, having experienced their first negative patient encounter. One possible explanation for differences in learning in a virtual environment including the lack of personalization between the student and the patient. Students may initially lack the skills necessary to pick up on nuanced information communicated through non-verbal means, such as a patient shifting in their chair translating to physical discomfort [25]. Students may have misinterpreted the seriousness of the patient’s injuries (spinal cord compression) secondary to her barely audible tone of voice; further, her symptoms of depression also may have overshadowed her pain. The findings with our student population agree with current research in the fields of prehospital stroke, psychiatric care, and palliative care where non-verbal cues provide critical information for the physician on the status of the patient [26,27,28]. Screening and diagnostic capabilities require enhanced skills in a virtual environment; many of these skills are not well established in student populations and must be learned with applied practice in a safe environment [29]. This may help explain why the patient from the charity care case is more difficult for students to connect with through a virtual environment compared to an in-person environment. We also considered the possibility of Zoom fatigue and overall wellbeing of students during the early stages of the pandemic; however, this does not account for the consistency of the effectiveness of the medical error case [30,31].

On balance, we found that attendance across all schools was greater after we transitioned to a virtual environment. The pandemic brought opportunities for reimagining and evaluating the effectiveness of the training of future healthcare professionals. We found we were able to tacitly deliver lessons in telehealth by reinforcing new and compelling ways of delivering simulation exercises. This is consistent with other research which has shown greater engagement resulting from increased flexibility and less formality when compared with in-person methodologies [32].

There are limitations to this study due to the nature of a pre-post and quasi-experimental design. The results presented may not be generalizable to students outside of the U.S., with a different curriculum, and with a different student population. Some of our students may have had prior training in interprofessional education or more experience in direct patient care, and this may have accounted for a lower change in pre-post scores.

## 5. Conclusions

Interactive simulations with interprofessional students in a healthcare environment are an excellent way to practice skills, make mistakes, and receive feedback for reflection in a safe environment. Faculty mentors from multiple disciplines serve as role models in debriefing sessions where students can ask questions and devote time to further inquiry. During the pandemic, we successfully transitioned two case-based simulations with a large number of students into a virtual environment. Further investigation into the reduced effect size for the virtual charity care case is needed, including additional qualitative feedback from the student learners. These details will help institutions that are considering virtual IPE and will provide more information to help them decide what cases and situations are appropriate, given the benefits related to scheduling/attendance but potential reductions in learning. A primer on telemedicine is recommended for all students in the health professions who intend to interact with patients, so they may learn informal cues, communication through videoconferencing modalities, and how to enhance their screening and diagnostic skills.

## Figures and Tables

**Table 1:** Demographics of students participating two in-person simulations from August 2019–May 2020. (*N* = 958 students).

Demographics	Total *N* = 479, (%) Medical Error	Total *N =* 479, (%) Charity Care
Professional School Affiliation		
Medical School	199 (41.55)	210 (43.84)
School of Nursing	184 (38.41)	184 (38.41)
Dental School	83 (17.33)	85 (17.75)
School of Public Health	13 (2.71)	0
* Professional/Research	0	0
Gender		
Female	322 (67.22)	333 (69.52)
Male	157 (32.78)	146 (30.48)
Age		
20–29	413 (86.22)	355 (74.11)
30–39	52 (10.86)	98 (20.46)
40	14 (2.92)	26 (5.43)

* Professional/Research, students included those from the School of Public Health, the School of Informatics, and the Graduate School of Biomedical Sciences.

**Table 2:** Demographics of students participating two virtual simulations from August 2020–May 2021. (*N* = 1013) students.

Demographics	Total *N* = 506, (%) Medical Error	Total *N* = 507, (%) Charity Care
Professional School Affiliation		
Medical School	192 (37.94)	198 (39.05)
School of Nursing	173 (34.19)	170 (33.53)
Dental School	122 (24.11)	115 (22.68)
School of Public Health	13 (2.57)	15 (2.96)
* Professional/Research	6 (1.19)	4 (0.79)
Gender		
Female	319 (63.04)	317 (62.52)
Male	187 (36.96)	190 (37.48)
Age		
20–29	430 (84.98)	372 (73.37)
30–39	54 (10.67)	108 (21.30)
40	22 (4.35)	27 (5.33)

Note: Percentages may not add up to 100 due to rounding. * Professional/Research, students in this category were from the Graduate School of Biomedical Sciences or School of Biomedical Informatics

**Table 3:** Effect sizes comparing the pre-test and post-test of the in-person simulation and the virtual simulation from August 2019 to May 2020, calculated as Cohen’s d for independent samples (Basic table).

Type of Simulation	In-Person Effect Size (95% CI)	Post-Tests Means above the Mean of the Pre-Test Mean *	Virtual Effect Size (95% CI)	Post-Tests Means above the Mean of the Pre-Test Mean *
Medical error (Inpatient)	0.84 (0.70–0.97)	80.0%	0.81 (0.68–0.94)	79.1%
Charity care (Outpatient)	0.84 (0.71–0.97)	80.0%	0.64 (0.51–0.77)	73.9%

* We assume the pre-test score is the control group and the post-test score is the experimental group.

## Data Availability

Data may be made available with reasonable request to the corresponding author.

## Supplementary Materials

The following supporting information can be downloaded at: https://www.mdpi.com/article/10.3390/healthcare10112184/s1, Supplement Material File S1: Questions from the interprofessional collaborative competency attainment survey (ICCAS) [19].
