# Supplementary material for: Unintended Consequences of a Transition to Synchronous, Virtual Simulations for Interprofessional Learners
Source: Healthcare (Basel). 2022 Oct 31;10(11):2184. doi: 10.3390/healthcare10112184 (PMC9691123; doi:10.3390/healthcare10112184)
Supplement: Supplementary file 1 [file healthcare-10-02184-s001.zip › healthcare-1890003-supplementary.pdf]

Supplement material File S1. Questions **from the interprofessional collaborative competency attainment survey (ICCAS) from Archibald et al, 2014.**

1. Promote effective communication among members of an interprofessional (IP) team
2. Actively listen to IP team members' ideas and concerns
3. Express my ideas and concerns without being judgmental
4. Provide constructive feedback to IP team members
5. Express my ideas and concerns in a clear, concise manner
6. Seek out IP team members to address issues
7. Work effectively with IP team members to enhance care
8. Learn with, from and about IP team members to enhance care
9. Identify and describe my abilities and contributions to the IP team
10. Be accountable for my contributions to the IP team
11. Understand the abilities and contributions of IP team members
12. Recognize how others' skills and knowledge complement and overlap with my own
13. Use an IP team approach with the patient to assess the health situation
14. Use an IP team approach with the patient to provide whole person care
15. Include the patient/family in decision-making
16. Actively listen to the perspectives of IP team members
17. Take into account the ideas of IP team members
18. Address team conflict in a respectful manner
19. Develop an effective care plan with IP team members
20. Negotiate responsibilities within overlapping scopes of practice
